# Clinical Criteria for Physician Aid in Dying

**DOI:** 10.1089/jpm.2015.0092

**Published:** 2016-03-01

**Authors:** David Orentlicher, Thaddeus Mason Pope, Ben A. Rich

**Affiliations:** ^1^ Center for Law and Health, Robert H. McKinney School of Law, Indiana University, Indianapolis, Indiana.; ^2^ Health Law Institute, School of Law, Hamline University, St. Paul, Minnesota.; ^3^ School of Medicine, University of California Davis, Davis, California.

## Abstract

More than 20 years ago, even before voters in Oregon had enacted the first aid in dying (AID) statute in the United States, Timothy Quill and colleagues proposed clinical criteria AID. Their proposal was carefully considered and temperate, but there were little data on the practice of AID at the time. (With AID, a physician writes a prescription for life-ending medication for a terminally ill, mentally capacitated adult.) With the passage of time, a substantial body of data on AID has developed from the states of Oregon and Washington. For more than 17 years, physicians in Oregon have been authorized to provide a prescription for AID. Accordingly, we have updated the clinical criteria of Quill, et al., based on the many years of experience with AID. With more jurisdictions authorizing AID, it is critical that physicians can turn to reliable clinical criteria. As with any medical practice, AID must be provided in a safe and effective manner. Physicians need to know (1) how to respond to a patient's inquiry about AID, (2) how to assess patient decision making capacity, and (3) how to address a range of other issues that may arise. To ensure that physicians have the guidance they need, Compassion & Choices convened the Physician Aid-in-Dying Clinical Criteria Committee, in July 2012, to create clinical criteria for physicians who are willing to provide AID to patients who request it. The committee includes experts in medicine, law, bioethics, hospice, nursing, social work, and pharmacy. Using an iterative consensus process, the Committee drafted the criteria over a one-year period.

## Introduction

More than 20 years ago, even before voters in Oregon had enacted the first aid in dying (AID) statute in the United States, Timothy Quill and colleagues proposed clinical criteria for AID.^1^ Their proposal was carefully considered and temperate, but there were little data on the practice of AID at the time. With AID, a physician writes a prescription for life-ending medication for a terminally ill, mentally capacitated adult. Consistent with the recommendation of the American Public Health Association, we use “aid in dying” rather than “physician-assisted suicide” to describe the practice.^2^

With the passage of time, a substantial body of data on AID has developed from the state of Oregon. For nearly two decades, physicians in Oregon have been authorized to provide a prescription for AID.^3^ Some data also come from Washington State, which enacted a statute in 2008 patterned after the Oregon law.^4^ Accordingly, for those physicians who are willing to provide AID, we have updated the clinical criteria of Quill, et al., based on these many years of experience.

To be sure, clinical criteria are included in the AID statutes in California, Oregon, Vermont, and Washington.^3–6^ But those criteria are incomplete. For example, while the states require physicians to ensure that the patient is making an informed and voluntary decision, the statutes provide insufficient guidance for physicians in their assessment of the patient's decision-making process. Our clinical criteria discuss the ways in which physicians should respond to a request for AID, including (1) discussion of the patient's reasons for requesting AID, (2) evaluation of the patient's decisional capacity, and (3) assessment of the patient's understanding of palliative measures that might be used instead of or concurrent with AID. In addition, while the statutes authorize the writing of a prescription for AID, they say nothing about the kinds or doses of medication that should be used. In contrast, our criteria provide specific recommendations for the prescriptions that physicians should write and the steps that patients should take in preparing their medication for ingestion.

Not only are statutory criteria incomplete in the states that have them, but criteria are wholly absent in other states. AID has been recognized as legitimate by courts in Montana and New Mexico.^7,8^ But, like the supreme courts of Colombia and Canada, those courts did so without issuing any guidelines, other than the requirement that patients be mentally capacitated adults who are terminally ill and able to self-administer the medication.^9,10^ Courts in several other states also could decide that no state law prohibits AID (as the Montana Supreme Court did) or that the state constitution guarantees a right to the practice (as found by a New Mexico trial court). Several such lawsuits were filed in early 2015.^11^ While AID remains ethically controversial, the pace of legalization is accelerating. Between January and September 2015, more than 25 state legislatures considered bills to authorize AID.^12^

With more jurisdictions authorizing AID, it is critical that physicians can turn to reliable clinical criteria. As with any medical practice, AID must be provided in a safe and effective manner. Physicians need to know (1) how to respond to a patient's inquiry about AID, (2) how to assess patient decision making capacity, and (3) how to address a range of other issues that may arise.

To ensure that physicians have the guidance they need, Compassion & Choices (the nation's oldest and largest nonprofit organization working to improve care and expand choice at the end of life [EOL]) convened the Physician Aid-in-Dying Clinical Criteria Committee, in July 2012, to create clinical criteria for physicians who are willing to provide AID to patients who request it. The committee includes experts in medicine, law, bioethics, hospice, nursing, social work, and pharmacy. Using an iterative consensus process, the committee drafted the criteria over a one-year period. The criteria draw upon over 25 combined years of extensive documentation and data collection from AID in Oregon and Washington, with the goal of supporting optimal patient care at EOL. Some statutory provisions impose requirements that are not necessary from a clinical perspective, so are not included. The full version of the clinical criteria can be found online. In the remainder of this article we provide a summary. (See online supplementary material at www.liebertpub.com/jpm.)

## Responding to Requests for AID

AID may be provided only to eligible patients—those with an incurable condition that will likely result in death within six months (or within a “relatively short time”).^13^ The patient also must be an adult resident of the state and possess the capacity to make major medical decisions.

A patient's request for AID must receive prompt evaluation. Physicians should explore the physical, psychological, spiritual, financial, and social issues influencing the request.^14^ The criteria include examples of questions that the physician may use to do this. The goals are (1) to deter any premature action by the patient, (2) to establish whether a request reflects decisional capacity and freedom from external pressure, and (3) to ensure that the patient is considering alternatives to AID. It is important that the physician identify patient concerns that could be addressed without AID. AID must reflect a considered and voluntary choice by the patient.

If the physician is concerned that a mental health condition may be impairing the patient's judgment or decisional capacity, the physician should refer the patient to a licensed psychiatrist or clinical psychologist for evaluation. A number of mental health screening assessments are available for physicians to use in the office. For example, the Patient Health Questionnaire (PHQ-9) is a validated instrument for detecting and diagnosing depression.^15^

## Ensuring Informed Consent

Studies show that few patients understand all their EOL options.^16^ Therefore, when a patient requests AID, the physician ought to explore with that patient the full range of EOL care available. First, patients should understand alternatives to hastening death: (1) hospice care and (2) aggressive management of symptoms, including deep sedation. Indeed, these two possibilities should be discussed with all patients in the terminal phase of disease. Patients who ask about AID should be referred for hospice care if they are not already enrolled. Furthermore, whenever feasible, the physician should obtain a second opinion from an experienced physician who ideally has palliative care experience. It is personally, professionally, and legally valuable for the physician to obtain this validation and confirmation. But in the exceptional cases in which it is infeasible to obtain a second opinion, that infeasibility should not preclude patient access to AID.^17^

Second, those patients who want to bring about a peaceful death at the time of their choosing should also understand that they may choose among several alternatives to AID: (1) discontinuing life-prolonging treatment, (2) palliative sedation to unconsciousness when indicated, and (3) voluntarily ceasing to eat or drink.^18^ The physician must assure the patient that that care will be provided to relieve any associated distress.

Physicians ought to encourage the patient both (1) to include family members in the patient's discussions of EOL care with the physician and (2) to discuss EOL planning with close relatives and loved ones. If a terminally ill patient worries that informing a family member would be problematic, the reasons for not informing must be fully explored and understood. Not only must the family make sense of the patient's death (if it occurs), but also the family may have insights into the motivations underlying the AID decision that are not obvious to the physician. It is recommended that a mental health professional or the physician conduct a family meeting to resolve these issues.

Physicians must thoroughly document the elements of an informed request for AID in the patient's medical record. These elements include patient understanding of diagnosis, prognosis, and the alternatives to AID. Physicians should also document that the patient understands (1) the near certainty that ingesting the prescribed life-ending medication will cause death; (2) the possibility that ingesting the medication could cause nausea or vomiting or, rarely, could fail to cause death; (3) that the patient always retains the right to decide against AID; and (4) that the physician is willing to continue caring for the patient and to address subsequent palliative needs, whether or not the patient chooses to take the medication.

Physicians must also inform patients about the self-administration requirement for AID. Patients must be capable of taking the medication on their own, usually by drinking from a cup but also by pouring through a feeding tube. Family or others may assist the patient by mixing the medication into a drink.

## Providing a Prescription

Oregon, Washington, and Vermont require a 15-day waiting period between the first request for AID and the writing of a prescription. When a waiting period is not required by state law, physicians may know the patient well enough to determine without difficulty that the request is voluntary, rational, and enduring. If physicians are uncertain about this, they should schedule a follow-up visit in 10 to 15 days to revisit the request. Putting a time buffer between requests and prescription writing generally will clear up any residual doubts. The attending physician also should encourage a meeting with the patient and family together to address any concerns about the patient's request.

Once physicians have written a prescription for life-ending medication, they must alert the patient's pharmacist. This allows the pharmacist to decide whether or not to participate and to have the appropriate medication available for pick-up.

In many cases in which a patient has received a prescription—more than one-third in Oregon—the patient never ingests the medication and dies from progression of the terminal illness. But even for these patients, the option of AID is a valuable benefit. These and other AID patients realize an improvement in their quality of life from the sense of control that comes with mere receipt (not ingestion) of the prescription.^19^

## End-of-Life Medication Procedure

The medication protocol is a two-step procedure. First, the patient takes an antiemetic (e.g., metoclopramide or ondansetron). Forty-five to sixty minutes later, the patient ingests 9 g of a short-acting barbiturate (e.g., secobarbital or pentobarbital). The powdered barbiturate is mixed with a half cup of water into a slurry and consumed. The barbiturate must be consumed quickly, within 30 to 120 seconds. Otherwise, the patient may fall asleep before ingesting an effective dose. The patient may then drink juice or other liquid as desired. The patient should not consume fatty foods within four to six hours prior to taking the medication.

Patients may wish to have their physicians present when they take their medication. This ought to remain a matter between patient and physician. It usually is a good idea for family members or friends to be with the patient at the time of ingestion to provide comfort. Indeed, a gathering of family and friends can be a rich experience for all.^20^ When a physician is not present, family or friends can notify the patient's physician, hospice, or funeral home of the time of death. Those present should understand that it is not necessary to call 911 when the patient goes into a coma and subsequently dies.

To maintain confidentiality of the patient's EOL decisions, physicians in Oregon and Washington indicate on the death certificate either “respiratory failure” or the patient's underlying terminal illness as the immediate cause of death. The manner of death is recorded as “natural.” This notation is similar to that used on death certificates following removal of a ventilator.^21^

## Conclusion

Although AID has received legal recognition only since 1997 and only in a few states, the experience to date permits the drafting of clinical criteria to guide physicians when their patients request AID. For physicians who are willing to provide AID, it is important that they be medically knowledgeable doing so. These criteria are designed to provide that knowledge and guidance.

## Supplementary Material

Supplemental data
