# Supplementary material for: Clinical Criteria for Physician Aid in Dying
Source: J Palliat Med. 2016 Mar 1;19(3):259–62. doi: 10.1089/jpm.2015.0092 (PMC4779271; doi:10.1089/jpm.2015.0092)
Supplement: Supplemental data [file Supp_Data.pdf]

Supplementary Material

Clinical Criteria for Physician Aid in Dying

Overview

IN JULY OF 2012, the Physician Aid-in-Dying Clinical Criteria Committee[a] met to create clinical criteria for physicians who are willing to provide aid in dying (AID) to patients who request it. The committee, which was convened by Compassion & Choices,[b] was comprised of experts in medicine, law, bioethics, hospice, nursing, social work, and pharmacy. To the extent possible, the committee developed these clinical criteria guided by the standards proposed by the Committee on Standards for Developing Trustworthy Clinical Practice Guidelines described in the publication by the Institute of Medicine Board on Health Care Services, *Clinical Practice Guidelines We Can Trust*.[1]

These clinical criteria draw upon 18 years' experience, including extensive documentation and data collection from Death with Dignity practice in the states of Oregon and Washington.[2–4] The goal is to support optimal patient care at the end of life (EOL), as well as provide respect for patient values, goals, and concerns in pursuit of a peaceful death. The Physician Aid-in-Dying Clinical Criteria Committee recommends physicians adopt these clinical criteria to guide their practice of AID.

Part 1. Abbreviations, Definitions, and Key Points

Abbreviations

AID, aid in dying; DWDA, Death with Dignity Act; EOL, end of life; POLST, physician/provider orders for life-sustaining treatment; QOL, quality of life; VSED, voluntarily stopping eating and drinking.

Definitions

Aid in Dying. This is the practice of a physician writing a prescription for life-ending medication for a terminally ill adult patient with decisional capacity.[5,6] Although AID has been referred to as ''physician-assisted suicide,'' this term is regarded as both inaccurate and pejorative by a growing number of medical and health organizations.[7,8] In jurisdictions in which the provision of a lethal prescription at the request of a terminally ill patient with decisional capacity has not been specifically authorized by statute or legal decision, physicians should consult with an attorney knowledgeable in such matters in order to assess the risks involved in providing this EOL option to a patient. It is of import to reiterate that many legal and clinical professionals assert that a dying patient's choice for a peaceful death differs fundamentally from suicide.[8–10]

Death with Dignity Act. These encompass the Oregon, Washington, and Vermont statutes that address physician AID.[2–4] A recent study published in the *New England Journal of Medicine* well describes the successful implementation of AID into the Seattle Cancer Care Alliance, the site of care for the Fred Hutchinson–University of Washington Cancer consortium, with a conclusion stating, ''Overall, our Death with Dignity program has been well accepted by patients and clinicians.''[11]

Decision making capacity (decisional capacity). This is the clinical term for a patient's ability to make informed health care related decisions. Decisional capacity is determined by a physician, but may be verified by a mental health professional if there is uncertainty. It refers specifically to a patient's ability to understand relevant information and their own clinical status, process information rationally and in accordance with their values, and make and communicate choices. By contrast, the legal term ''mental competence'' is determined by the courts.[12,13]

Key points

Before describing the clinical criteria, three key points will be discussed.

Physician's role. The role of the physician in the care of a patient with terminal disease, following a complete assessment, is to offer as much information as the patient may wish concerning (1) the nature of their condition; (2) its prognosis with and without available clinical interventions; (3) hospice and palliative care options; and (4) social,

---

[a]The Physician Aid-in-Dying Clinical Criteria Committee: Robert Brody, MD, University of California San Francisco, San Francisco; David Muller, MD, New York; Ben Rich, JD, PhD, University of California Davis School of Medicine Alumni Association Endowed Chair of Bioethics, Davis, CA; Thaddeus Mason Pope, JD, PhD, Director of the Health Law Institute, Hamline University School of Law, St. Paul, MN; Ann Jackson, MBA, Former Executive Director of Oregon Hospice Association, Portland; Susan Shapiro, MSW, Geriatric Care Manager, AZA Care Management and Home Care, Boston; Lisa Anderson, DrPH, MA, MSN, Director, Clinical Ethics Consult Service, Assistant Clinical Professor, University Illinois Medical Center, Chicago; E. James Lieberman, MD, MPH, Clinical Professor of Psychiatry, Emeritus, George Washington University School of Medicine, Washington DC.; Don Downing, RPh, Professor, University of Washington School of Pharmacy, Seattle; David Orentlicher, MD, JD, Co-director, William S. and Christine S. Hall Center for Law and Health, Indianapolis; Robert Wood, MD, Clinical Professor of Medicine, University of Washington, Seattle; Thomas Preston, MD, Professor of Medicine, University of Washington, Seattle; and Judy Neall Epstein, ND, Medical Director, Compassion & Choices.

[b]Compassion & Choices is the nation's oldest and largest nonprofit organization working to improve care and expand choice at EOL. It has been the leader of the EOL choice movement for more than 30 years through support, education, and advocacy.

psychological, and spiritual support available to the patient and family. Patients who express an interest in, or desire for, controlling the timing and manner of death with a lethal prescription should be carefully assessed consistent with these clinical criteria and applicable state laws or regulations where they exist.

Eligibility criteria. To qualify for AID a patient must:

1. ***Be terminally ill.*** The patient must be medically diagnosed with an incurable or irreversible condition that will, in the opinion of the physician, likely result in death within a six-month period. This six-month standard has been adopted in Oregon, Washington, and Vermont statutes for AID. In contrast, Montana law stipulates only that the patient's death be likely to occur within a relatively short time to be eligible for AID.[14–16] It is recommended that patients who ask about a lethal prescription in any state be referred for hospice care and support, if they are not already enrolled. Being enrolled in hospice generally represents a six-month or less prognosis and provides excellent care for the patient and family. However, note that patients who are admitted to hospice are being admitted for hospice services only and not for AID.
2. ***Be an adult (18 years of age or older) resident of the state.***
3. ***Have decisional capacity.*** Candidates for AID must be capable of making health care decisions. In general, most patients are presumed to have the capability to make health care decisions unless their presentation raises doubt. However, the prescribing physician should screen the patient to rule out untreated acute or chronic depression, or another mental condition impairing decisional capacity. Note that the presence of physical, mental, or developmental disorders does not necessarily imply loss of decisional capacity.[17]

Recordkeeping and documentation of informed request. The physician should thoroughly document the elements of an informed request for AID in the patient's chart. These elements include patient awareness and understanding of:

1. Diagnosis
2. Prognosis
3. The near certainty that ingesting the prescribed life-ending medication will cause death
4. The possibility that ingesting the medication could cause nausea or vomiting or, rarely, could fail to cause death, allowing the patient to return to consciousness
5. Alternatives to life-ending medication
6. The right to rescind their request for AID medication or decline to ingest it

The patient should understand that neither making a request for AID nor filling a prescription for life-ending medication obligates him or her to ingest it. In addition, the physician should document the form of request for AID by the patient (verbal or written), and evidence the patient has decisional capacity and is acting voluntarily.

Note: Although the DWDAs of Oregon, Washington, and Vermont require documentation of numerous specific elements of care during evaluation for AID, the consensus of the committee is that those listed above are adequate for physicians providing AID outside those three jurisdictions. It is important to note that for many patients who ultimately receive and fill a prescription for life-ending medication, just having it available as an option provides peace of mind that enables them to allow their dying process to unfold naturally. Many who have the medication on hand never take it (see Table 1).

Part 2. Specific Clinical Criteria for Physician Aid-in-Dying and Prescribing Responsibilities

Physician evaluation

Exploring the basis of a request for AID. A patient's request to end their life during terminal illness, whether by AID or an alternative method, should receive prompt evaluation by the physician. The goals of this evaluation are (1) to deter a patient's premature or violent action; (2) to provide psychiatric intervention if a request is irrational; (3) to establish that a request reflects decisional capacity and freedom from external pressure (coercion); and (4) to ensure that the patient is aware of alternatives such as hospice, comfort (palliative) care, and/or appropriate medical treatments. The physical, psychological, spiritual, financial, and social issues that have influenced the request for AID should be explored in order to determine that the patient is acting voluntarily.[17–20] Table 2 shows the most frequently reported concerns of patients who received a prescription for life-ending medication in Oregon.[21]

Patient must act on own initiative. Patients who request AID and plan to take life-ending medication must be educated about and prepared to self-administer the drug. As an example, Oregon's Death with Dignity law explicitly supports patient autonomy and recognizes, for example, that ingesting EOL medication by swallowing, sucking on a straw, or sipping from a cup qualifies as self-administration.[22]

Explore alternatives to medication. Studies show that few patients understand all of their EOL options.[23] However, effective communication among patient, family, and health care providers that begins at the time of diagnosis of a terminal illness or disease improves outcomes for both patient and families.[24] Therefore, with the patient requesting AID—and possibly the family—the physician should explore the full range of EOL choices available. These could include but are not limited to consideration of referral to a palliative care specialist who is experienced with managing patients during progressive illness and changes in care.[25]

Table 1. Comparison of the Number of Patients Dispensed EOL Medication and the Number of Patients Who Died from Ingesting EOL Medication

State	*Oregon*[26]	*Washington*[27]
Persons dispensed EOL medications	1327	549
Persons dying of EOL medications	859	323

Table 2. Reasons for Wanting Physician Aid in Dying

Reason	*Median score (IQR)*[a]
Wanting to control circumstances of death	5 (4–5)
Future poor quality of life	5 (4–5)
Future pain	5 (4–5)
Future inability to care for self	5 (3–5)
Loss of independence	5 (3–5)
Wanting to die at home	5 (1.25–5)
Perception of self as burden	4 (2–5)
Loss of dignity	4 (1–5)
Witnessed bad death(s)	4 (1–5)
Future mental confusion	3.5 (1–5)
Not wanting others to care for me	3 (1.25–5)
Worry about loss of sense of self	3 (1–5)
Future dyspnea	3 (1–5)
Ready to die	3 (1–5)
Future fatigue	3 (1–5)
Future loss of bowel/bladder control	3 (1–5)
Unable to pursue pleasurable activities	1 (1–4)
Life is pointless	1 (1–4)
Life tasks are complete	1 (1–4)
Perceive self as financial drain	1 (1–3.75)
Poor quality of life	1 (1–3)
Current pain	1 (1–2.75)
Unable to care for self now	1 (1–2)
Current dyspnea	1 (1–2)
Lack of support	1 (1–1)
Depressed mood	1 (1–1)
Current mental confusion	1 (1–1)
Current loss of bowel/bladder control	1 (1–1)

[a]Participants marked the importance of their reasons for requesting physician AID on a scale from 1 to 5: 1 was ''reason not at all important in decision to request a lethal prescription'' and 5 was ''reason is very important in decision to request a lethal prescription.'' Marks not on an integer were estimated to the quarter interval.

AID, aid in dying; IQR, interquartile range (25th percentile to 75th percentile).

Other measures often employed include (1) hospice or other comfort care, (2) aggressive pain and/or symptom management, and (3) palliative sedation (which may bring about death through dehydration if parenteral fluids are withheld). These options should be discussed with all patients who have terminal disease regardless of whether a request for AID has been made. Also, the patient should be informed whether their medical community can offer EOL care from any one of a number of places such as home, hospital, hospice, or an assisted living center. Finally, the patient should understand that he or she may choose a way other than life-ending medication to bring about a peaceful death at the time of his or her choosing. This could include voluntarily stopping eating and drinking (VSED) or foregoing or discontinuing life-prolonging treatment (e.g., implanted cardiac device, dialysis, feeding tube, ventilator).

Examples of questions that the physician may use to explore whether the patient has considered all EOL options are[17]:

(1) What is most troubling about your current symptoms, care, or treatment, and what might help you to be more comfortable?
(2) Have you ever thought about what a ''good death'' would look like for you?
(3) Where would you prefer to spend your final days, and where would you like to die?

It is important that the physician check for and address any symptoms or other medical conditions that could influence the patient's request for life-ending medication (e.g., unmanaged pain or other treatable symptoms). Whenever feasible, the physician should obtain a second opinion from an experienced physician who ideally has palliative care experience. It is personally, professionally, and legally valuable for the physician to obtain this validation and confirmation. But in the exceptional cases in which it is infeasible to obtain a second opinion, that infeasibility should not preclude patient access to AID.

Evaluation for conditions that can diminish decisional capacity. If the physician is concerned that the patient may have a mental health condition that is impairing their judgment or decisional capacity, the physician should refer the patient to a licensed psychiatrist or psychologist for evaluation. Approximately 5% of patients who have completed a request for AID under the Oregon and Washington AID statutes were referred for a psychiatric or psychological evaluation.[26,27]

A variety of conditions can diminish a patient's decisional capacity, including delirium, dementia, psychosis, CNS disease involvement, substance abuse, depression, or mania. However, situational depression is not uncommon in terminally ill patients and does not in itself render the patient ineligible for AID.[17,28] The physician should delay writing a prescription for life-ending medication until the results of any requested psychiatric or psychological evaluation are available and the patient's decisional capacity is confirmed. The patient may benefit from consulting a therapist, psychologist, or social worker if he or she appears to be making a request for AID due to overwhelming psychosocial issues (e.g., fear of further suffering or pain, feeling a burden to the family and society, sense of loss of dignity or autonomy, financial issues) that are affecting decision making.[17,18]

A number of brief mental health screening assessments are available for primary care physicians to use in the office. The Oregon DWDA Guidebook recommends that all patients who request AID under the state's statute be screened for depression with a validated instrument, such as the Patient Health Questionnaire (PHQ-9).[29] This nine-item depression scale can be administered and scored by a primary care clinician and/or office staff. Further discussion of the PHQ-9 can be found at the MacArthur Initiative on Depression and Primary Care website.[28,30] Special recommendations for psychologists and psychiatrists evaluating terminally ill adults under the DWDA of either Washington or Oregon have been provided by the Washington State Psychological Association (WSPA).[28,29] These recommendations may also be useful to psychologists and psychiatrists outside these jurisdictions in evaluating candidates for AID.

Advance directive and physician/provider orders for life-sustaining treatment. Candidates for AID should understand that due to their disease process they could lose either their decisional capacity or their ability to self-administer

medication and thus their eligibility for AID. Therefore, the patient who has not completed an advance directive or has not requested a Physician/Medical Orders for Life Sustaining Treatment (POLST or MOLST) from either a physician or a physician's representative should consider doing so. Through an advance directive, the patient documents decisions regarding medical care and identifies a surrogate decision maker to represent him or her in the event of decisional incapacity.[20]

An advance directive can also be useful in the unlikely event the patient fails to die after ingesting a lethal dose of medication for AID. Here, the directive would inform the physician about preferred care if the patient can't speak for himself and whether, for example, the patient would wish to proceed to palliative sedation or be allowed to fully regain consciousness. Overall, the percentage of patients regaining consciousness after ingesting EOL medication in Oregon and Washington has been 0.4%.[26,27]

Additional issues to consider

Implanted cardiac devices. Patients with an implanted cardioverter-defibrillator should be informed of the option of turning it off to avoid receiving shocks when their heart fibrillates or stops. Demand pacemakers may be adjusted to the lowest possible setting prior to ingestion of a lethal dose of medication pursuant to these clinical criteria. As the heart slows, the pacemaker may still fire, but it is unlikely to produce effective heart beats or prolong the dying process. Patients may need assistance from the physician in making these arrangements with the appropriate persons. Patients who are completely pacemaker dependent may not be able to tolerate these adjustments, as they may lose consciousness when the pacemaker settings are adjusted downward. There is currently a strong consensus among health care professionals that deactivation of implantable cardiac devices (CIEDs) is governed by the same ethical and legal principles as other forms of life-sustaining interventions and therefore can be performed at the patient's request.

Assessment of decisional capacity at time of ingestion. If the patient decides to ingest the medication, assessment of decisional capacity immediately prior to ingestion is desirable. If the physician or other medical personnel will not be present at the time of ingestion, the family may be advised to determine that the patient is fully aware that ingesting the medication will cause their death. To accomplish this, the physician or family could ask the patient, ''What is your understanding of what will happen when you take this medication?'' A reply such as ''I will die'' suggests that the patient's decisional capacity is intact.

Waiting period. Generally, a physician who receives a request for AID has formed a relationship with that patient and will be able to determine without difficulty if the request is voluntary, rational, and enduring. However, occasionally there may be a concern that this is not the case. In such an instance, the physician should schedule a follow-up visit in 10 to 15 days to revisit the request. Putting a time buffer between a request and prescription-writing generally will clear up any confusion in this regard. The attending physician should also consider meeting with the patient and their family members or life partner to evaluate any concerns and alleviate family member concerns.

Statutes in Oregon, Washington, and Vermont currently require a 15-day waiting period between the day that the patient first makes an oral request for AID and the day that the physician may prescribe the life-ending medication (following a second oral request). The median duration of time between first request for AID and death is 47 days (range 0–1905) in 859 patients who received AID by 2014 in Oregon since the program's inception in 1998. This finding suggests that a waiting period under these circumstances is possible for most patients.[26] However, the committee believes recommending a mandatory 15-day waiting period exposes some patients to unnecessary and intolerable pain and suffering, and is not required in all cases. This can be left to physician discretion.

Family involvement. It is highly recommended that the patient discuss their EOL plans with close relatives and loved ones. If a terminally ill patient worries that informing a family member would be problematic, the reasons for not informing must be fully explored and understood. Not only must the family make sense of the patient's death (if it occurs), but also the family may have insights into the motivations underlying the AID decision that are not obvious to the physician. It is recommended that a mental health professional or the physician conduct a family meeting to resolve these issues. The patient should also consider planning to ingest life-ending medication in the presence of a family member, a close friend, a significant other, a spiritual or religious advisor, and/or the physician or a clinician experienced in working with families. A gathering of family and friends sitting with their loved one at this time can be a rich experience for all.

End-of-life medication procedure

Medication. The medication protocol is a multistep procedure. The first step consists of the ingestion of an antiemetic (metoclopramide 20 mg or ondansetron 4–8 mg or prochlorperazine 20 mg) followed 45 to 60 minutes later by 9 g of short-acting barbiturate, e.g., secobarbital or pentobarbital. The powdered medication is mixed with a half cup of water into a slurry and then consumed. It is important that the medication be entirely consumed quickly, within 30 to 120 seconds. Otherwise, sleep may overtake the patient before an effective dose is ingested. The patient may then drink juice or some other liquid as desired. The patient should not consume fatty foods within four to six hours prior to taking the medication. The prescription for the barbiturate is written for #90 100 mg, with no refills, and ''take as directed.''

Coordinating with pharmacy. Once the physician has written the prescription for life-ending medication, he or she should alert the patient's pharmacist. This allows the pharmacist to have the appropriate medication available when the patient arrives to pick it up. (It is often not a stocked medication and may need to be ordered.)

Patient's management of their medication. Although some patients fill their prescription immediately after their physician provides it, many delay doing so. Reasons patients

delay filling their prescription vary. Some wait until their suffering becomes unbearable. Others wait in order to put off the significant expense of the medication until they are sure they are going to use it. However, many patients who obtain medication and never ingest it derive significant comfort and peace of mind just from having it available.[31] Data from Oregon and Washington AID support this finding.[26,27]

Inability to ingest medication. Patients who are unable to self-administer the medication must consider other options if they choose to advance the time of their death. Also, patients who are unable to take the entire amount of medication by mouth in less than two minutes, or those with poor absorption or poor ability to take anything by mouth due to gastrointestinal difficulties, are not candidates for ingestion. Some patients who have lost the ability to swallow but have a feeding tube in place may self-administer the medication via the tube.

Patient to inform physician of plan. The patient should understand it is important that they inform the physician about their plan for taking the life-ending medication. It is helpful for the patient to be in hospice, not only to obtain comfort care but also to alert others that the patient's death is expected. A perceived unexpected death could jeopardize the patient's confidentiality and cause undue distress for the grieving family.

Hospices in Oregon and Washington have become more supportive of AID over the past 18 years of open practice in those states. Their support of AID is along a broad continuum extending from being as uninvolved as possible to being significantly supportive of patients and having hospice staff present at the time of death.[32]

Physician attendance at death. The patient may prefer to have his or her physician present for their own reassurance when they die. This should remain a matter between patient and physician. If the physician cannot be present, it is a good idea to be sure someone will be with the patient at the time of death so that hospice can be notified of the death.

How terminally iii patients die after self-administering lethal medication

The 2015 Oregon Department of Human Services 17th annual report indicates that since passage of Oregon's DWDA in 1997, a total of 1327 people have received DWDA prescriptions. Of those, 859 died from ingesting a lethal quantity of barbiturate (98.9%) or barbiturate plus morphine (1.2%).[26] All patients who died entered a coma within 1 to 38 minutes (median 5 minutes) and died within 1 minute to 104 hours (median 25 minutes) after ingesting the quantity of barbiturate.[26]

Notification to hospice and physician of death. After the patient dies, a family member, friend, significant other, or professional present at the patient's death will notify hospice and the physician of the death, making note of the time of death. If the deceased was not in hospice at the time of death, the designated funeral home or mortuary should be notified. The family should understand that it is not necessary to call 911 when the patient goes into a coma and subsequently dies.

Death certificate. To maintain confidentiality of the patient's EOL decisions, the physician should indicate on the death certificate either that ''respiratory failure'' or the patient's underlying terminal illness was the immediate cause of death. The manner of death can be recorded as ''natural.'' This choice of notation is similar to that used on death certificates in death following removal of a ventilator.[33]

References

1. Graham R, et al.: *Clinical Practice Guidelines We Can Trust*. Washington, DC: The National Academies Press, 2011.
2. Or Rev Stat §§127.800–127.897.
3. Vt Stat Ann Tit 18, §§5281–5292.
4. Wash Rev Code §§70.245.010–70.245.904.
5. American Public Health Association: Patients' rights to self-determination at the end of life. www.apha.org/advocacy/policy/policysearch/default.htm?id=1372. American Public Health Association, 2008. (Last accessed October 22, 2015).
6. American Medical Women's Association: Position paper on aid in dying. American Medical Women's Association, 2007. www.amwa-doc.org/wp-content/uploads/2013/12/Aid_in_Dying1.pdf. (Last accessed October 22, 2015.)
7. Quill TE, Cassel CK, Meier DE, et al.: Care of the hopelessly ill: Proposed clinical criteria for physician assisted suicide. N Engl J Med 1992;327:1380–1384.
8. Tucker K: At the very end of life: The emergence of policy supporting aid in dying among mainstream medical & health policy associations. Harv Health Policy Rev 2009;10:45–47.
9. Faberman RK: Terminal illness and hastened death requests: The important role of the mental health professional. Prof Psychol Res Pr 1997;28:544–547.
10. Bostwick JM, Cohen LM: Differentiating suicide from life-ending acts and end-of-life decisions: A model based on chronic kidney disease and dialysis. Psychosomatics 2009; 50:1–7.
11. Loggers ET, et al.: Implementing a death with dignity program at a comprehensive cancer center. N Engl J Med 2013;368:1417–1424.
12. Snyder L, Leffler C; American College of Physicians Ethics, Professionalism, and Human Rights Committee: *American College of Physicians Ethics Manual, 6th ed.* Ann Intern Med 2012;156:73–104.
13. Ganzini L, Volicer L, Nelson WA, et al.: Ten myths about decision-making capacity. J Am Med Dir Assoc 2004;5: 263–267.
14. Mont Code Ann §50-9-102(16).
15. *Baxter v State of Montana,* 224 P.3d 1211 (2009).
16. Montana Board of Medical Examiners: Physician aid in dying, 2011. bsd.dli.mt.gov/license/bsd_boards/med_board/pdf/Final%20Draft%20Physician%20Aid%20in%20Dying%20Statement.pdf. (Last accessed March 24, 2012.)
17. Vazquez LD, Santone MD: Psychosocial end-of-life considerations for healthcare providers. Ochsner J 2011;11: 317–324.
18. Werth JL, Gordon JR, Johnson RR: Psychosocial issues near the end of life. Aging Ment Health 2002;6:402–412.
19. Hudson PL, Schofield P, Kelly B, et al.: Responding to desire to die statements from patients with advanced disease: Recommendations for health professionals. Palliat Med 2006;20:703–710.
20. Back AL, Anderson WG, Bunch L, et al.: Communication about cancer at the end-of-life. Cancer 2008;113:1897–1910.

21. Ganzini L, Goy E, Dobscha S: Oregonians' reasons for requesting aid in dying, Arch Int Med 2009;169:489–492.
22. Or Rev Stat §127.505.
23. Silveira MJ, DiPiero A, Gerrity MS, Feudtner C: Patients' knowledge of options at the end of life: Ignorance in the face of death. JAMA 2000;284:2483–2488.
24. Wright AA, Zhang B, Ray A, et al.: Associations between end-of-life discussions, patient mental health, medical care near death, and caregiver bereavement adjustment. JAMA 2008;300:1665–1673.
25. Morrison RS, Meier DE: Palliative care. N Engl J Med 2004;350:2582–2590.
26. Oregon Public Health Division: Oregon's Death with Dignity Act 2015. public.health.oregon.gov/ProviderPartnerResources/EvaluationResearch/ DeathwithDignityAct/Documents/year17.pdf. (Last accessed October 22, 2015.)
27. Washington State Department of Health: 2014 Death with Dignity Act Report. Washington State Department of Health, 2015. www.doh.wa.gov/portals/1/Documents/Pubs/422-109-DeathWithDignityAct2014.pdf. (Last accessed October 22, 2015.)
28. Washington State Psychological Association: The Washington Death with Dignity Act: WSPA Guidelines for Mental Health Professionals. Washington State Psychological Association, 2009. www.wapsych.org/associations/2046/files/DWD%20Guidelines%206-3-09.pdf. (Last accessed October 22, 2015.)
29. Dunn P, Reagan B (eds); Task Force to Improve the Care of Terminally-Ill Oregonians: *The Oregon Death with Dignity Act: A Guidebook for Health Care Professionals, 2008e.* Portland, OR: The Center for Ethics in Health Care, Oregon Health & Science University, 2008. www.ohsu.edu/xd/education/continuing-education/center-for-ethics/ethics-outreach/upload/Oregon-Death-with-Dignity-Act-Guidebook.pdf. (Last accessed October 22, 2015.)
30. MacArthur Initiative on Depression & Primary Care at Dartmouth & Duke: Depression Management Toolkit. 2009. www.integration.samhsa.gov/clinical-practice/macarthur_depression_toolkit.pdf. (Last accessed October 22, 2015.)
31. Sowers W: Physician aid in dying and the role of psychiatry. Psychiatr Times 2004:21:37–38.
32. Campbell CS, Cox JC: Hospice and physician-assisted death: Collaboration, compliance, and complicity Hastings Center Rep 2010;40:26–35.
33. Tucker K: Aid in Dying: Guidance for an emerging end-of-life practice. Chest 2012:142:218–224.
